# Delayed bilateral spontaneous renal rupture after surgery for unilateral upper ureteral calculi: a case report

**DOI:** 10.3389/fmed.2023.1173386

**Published:** 2023-10-06

**Authors:** Wen Tang, Denghao Yang, Tao Wu, Guobiao Liang

**Affiliations:** Department of Urology, The Affiliated Hospital of Zunyi Medical University, Zunyi, China

**Keywords:** bilateral, delayed, spontaneous renal rupture, ureteral calculi postoperative, infection, case report

## Abstract

Spontaneous renal rupture is a rare clinical condition characterized by spontaneous bleeding in the renal subcapsular and perinephric spaces in patients without a history of trauma. It occurs mainly in pathologic kidneys and after some renal surgeries. We report a 40-year-old male patient admitted with a diagnosis of gallstones with cholecystitis due to fever and abdominal pain after unilateral ureteral calculi. The patient developed delayed right renal rupture hemorrhage during treatment, controlled after selective arterial embolization (SAE). Still, the patient developed spontaneous left renal rupture due to a systemic inflammatory response. Finally, the patient’s life was saved after several selective embolizations of the renal artery. We retrospectively analyzed this case to improve our understanding of the disease.

## Introduction

Spontaneous renal rupture is a rare urologic emergency defined as a rupture of the renal parenchyma, collecting system, or renal vessels (1). Since there is no history of trauma in cases of spontaneous renal rupture, there is usually an underlying renal disease, the leading causes of which are malignancy and vascular smooth muscle lipoma (2, 3). Other causes include vasculitis, diseases causing coagulopathy, infections, and anticoagulant use (4, 5). In addition, spontaneous renal rupture is seen after various kidney-related operations. Extracorporeal shock wave lithotripsy (ESWL) is a standard non-invasive treatment for different urinary stones. However, it may cause complications such as acute hematuria, perirenal hematoma, and chronic renal interstitium fibrosis, with the incidence of perirenal hematoma being about 4.6% (6–8). Ureteroscopic lithotripsy is often the preferred operation in clinical practice because of its precise positioning, safety, and effectiveness. Spontaneous renal rupture after the operation may be related to pathological changes in the patient’s kidney, intraoperative pelvic pressure elevation, sudden postoperative pelvic pressure decline, and postoperative infection (9). Clinically, spontaneous renal rupture occurs mainly in the unilateral kidney; spontaneous rupture of both kidneys is extremely rare, and only a few cases have been reported (10–13). This report describes a rare case of bilateral renal rupture and bleeding that occurred after ureteral calculi surgery, which is a guide to the etiology, diagnosis, and treatment of spontaneous renal rupture.

## Case presentation

**Figure 1:**
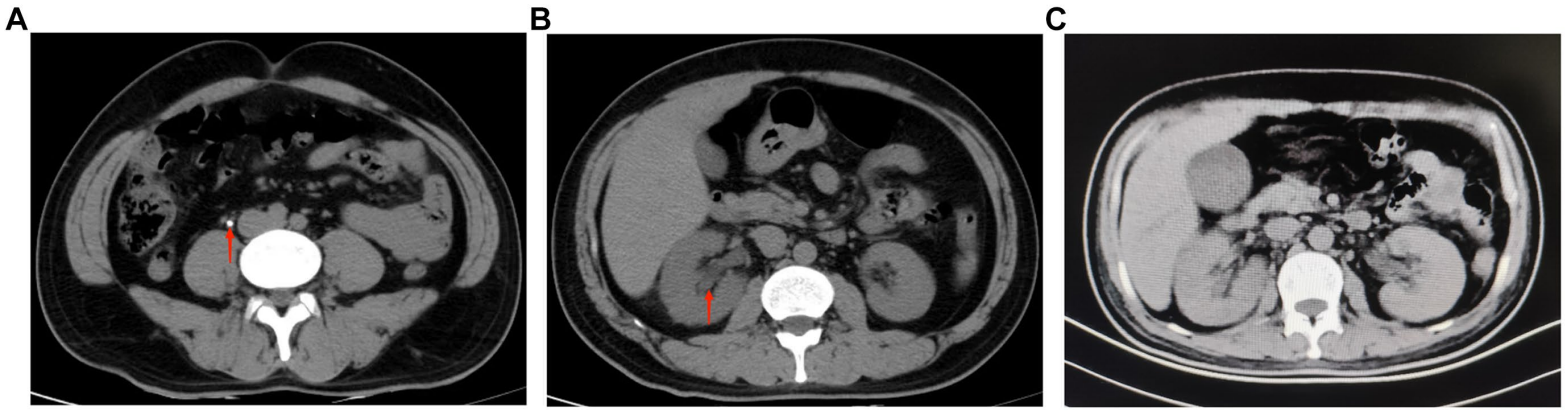
**(A)** Ureteroscopic lithotripsy preoperative abdominal CT showed a right upper ureteral stone (red arrow), about 0.5 cm in diameter (July 24, 2022). **(B)** Ureteroscopic lithotripsy preoperative abdominal CT showed no significant abnormality in the morphology, size and density of both kidneys, and clear perinephric space in both kidneys. The red arrow shows mild dilatation of the right renal pelvis (July 24, 2022). **(C)** The patient was transferred to our department for the first abdominal CT. The images showed no significant abnormalities in both kidneys and bilateral ureters (September 15, 2022, day 12 of admission).

**Figure 2:**
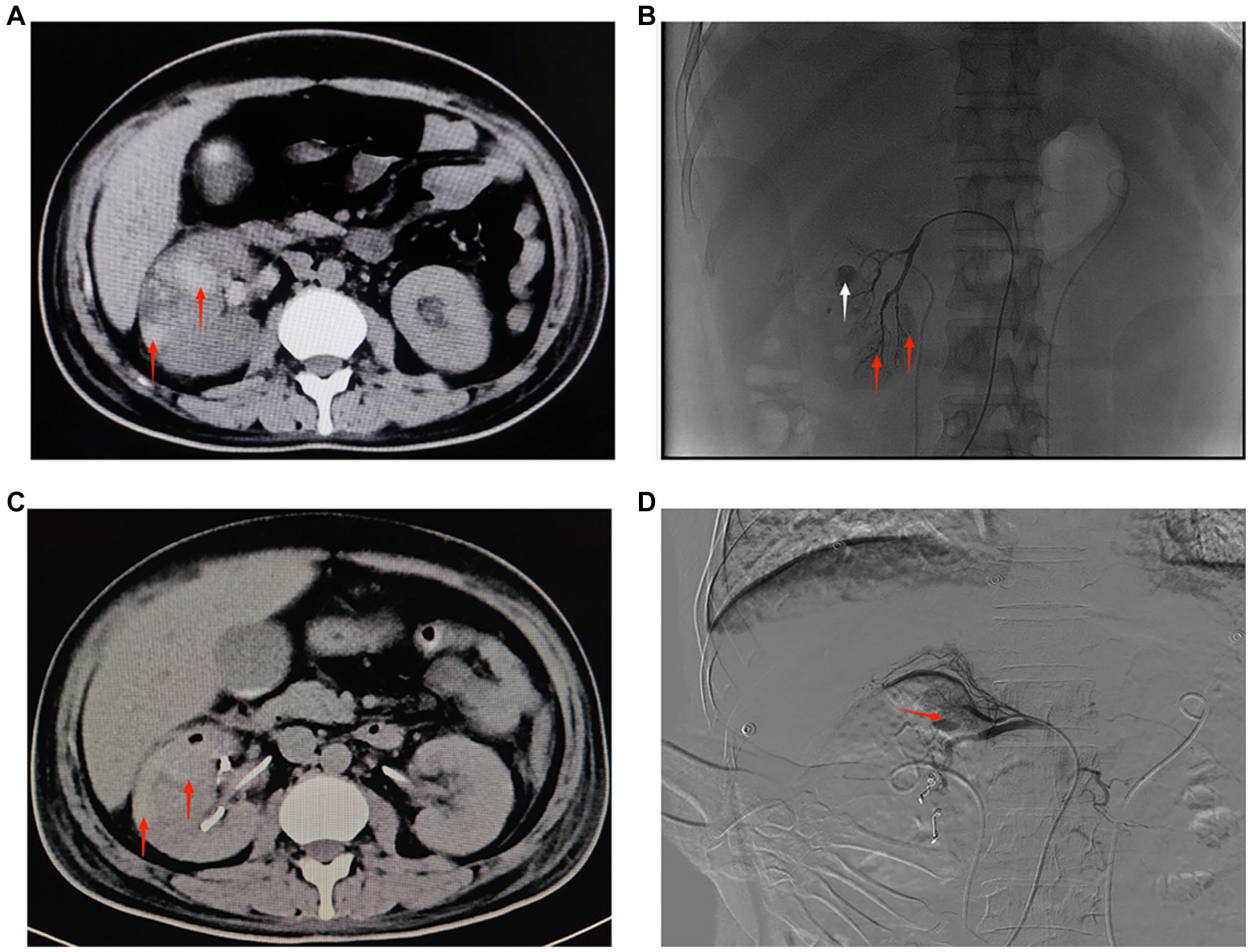
**(A)** CT of both kidneys. The right kidney is increased in size, heterogeneous in density, with multiple flaky hyperdense shadows, and clear perirenal space in both kidneys. The red arrow shows a hematoma in the lower pole of the right kidney (September 19, 2022, day 16 of admission). **(B)** Angiography of the right renal artery. Red arrows show active extravasation of multiple subsegmental arteries supplying the lower pole. White arrow indicates pseudoaneurysm formation (September 19, 2022, day 16 of admission). **(C)** CT of both kidneys. The right kidney is increased in size, heterogeneous in density, with multiple patchy hyperdense shadows, and clear perirenal space in both kidneys. The red arrow shows a hematoma in the upper pole of the right kidney (September 22, 2022, day 19 of admission). **(D)** Digital subtraction angiography of the right renal artery. Red arrow indicates active extravasation of the subsegmental artery supplying the superior pole (September 22, 2022, day 19 of admission).

**Figure 3:**
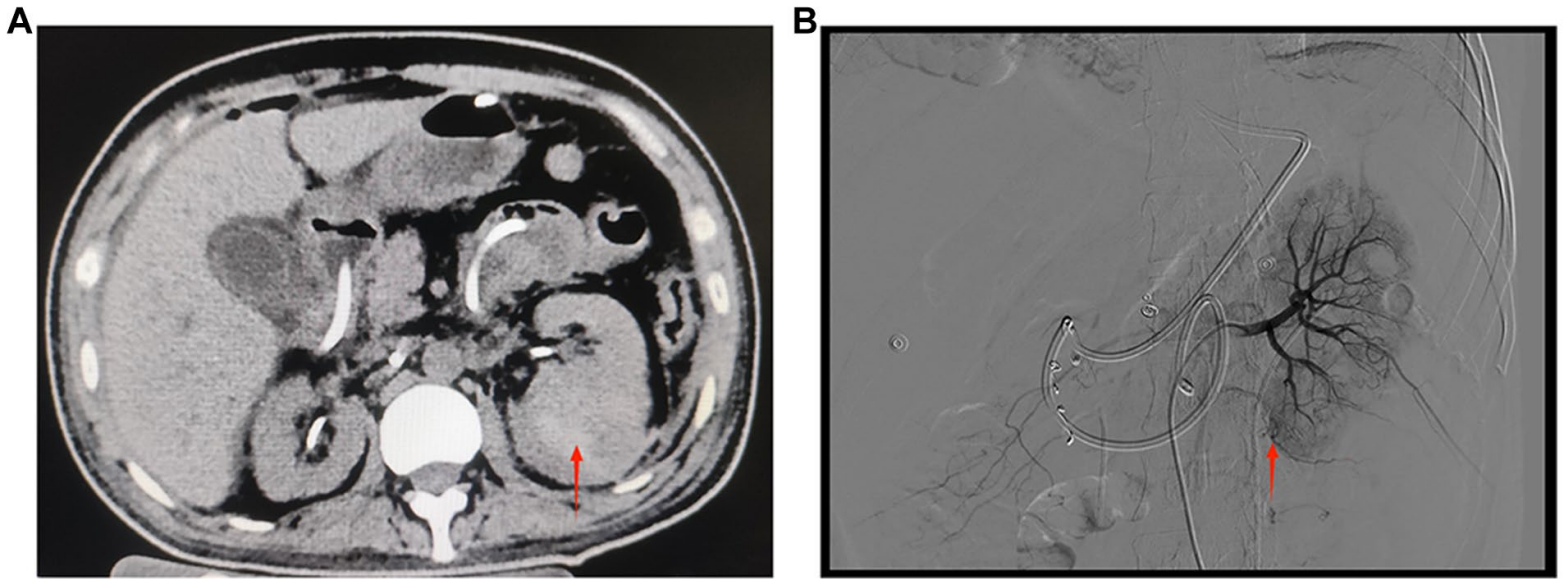
**(A)** CT of both kidneys. The left kidney is increased in size, heterogeneous in density, with multiple patchy hyperdense shadows and a few flocculent hyperdense shadows seen in the perirenal space of the left kidney. The red arrow indicates a hematoma in the lower pole of the left kidney (September 27, 2022, day 5 of transfer to ICU). **(B)** Digital subtraction angiography of the left renal artery. The red arrow shows suspicious active extravasation of the subsegmental artery supplying the lower pole (October 18, 2022, day 26 of transfer to ICU).

**Figure 4:**
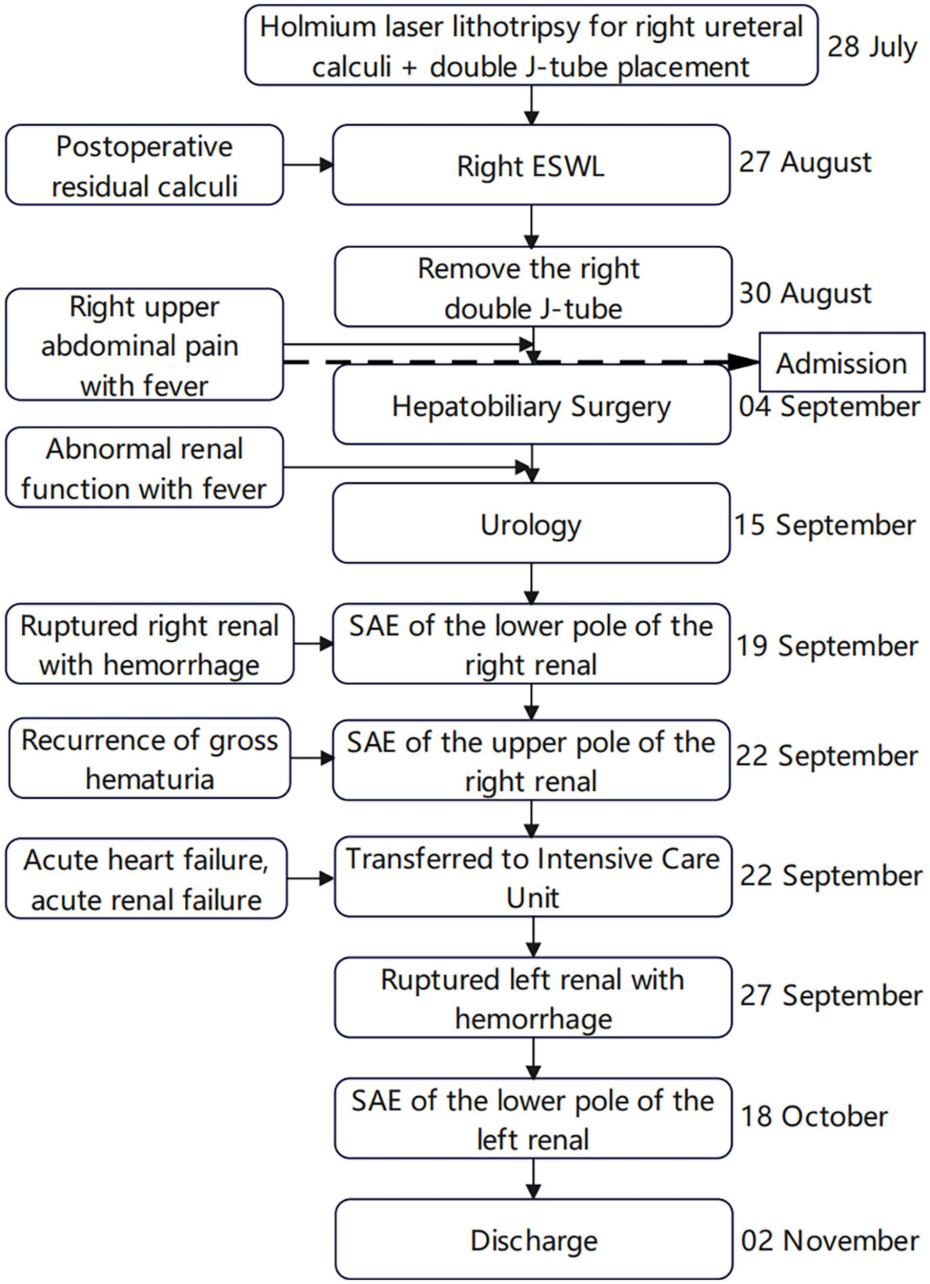
Timeline from the first surgery to discharge. All of these events occurred in 2022.

A 40-year-old male patient presented to our emergency department with right upper abdominal pain with intermittent fever for 5 days and was considered to have gallstones with cholecystitis after an abdominal ultrasound examination. After the patient was admitted to the Department of Hepatobiliary Surgery, the patient’s epigastric pain improved after 11 days of symptomatic treatment, but creatinine gradually increased, and fever still appeared intermittently. Therefore, the patient was referred to our department for further treatment of abnormal renal function and fever. The patient had no history of underlying disease, hypertension, diabetes, hematological system, family history of hereditary diseases, and trauma. One month before admission, he underwent a holmium laser lithotripsy and right double J-tube placement in a local hospital for right upper ureteral calculi and hydronephrosis (Figures 1A,B). The operation went successfully. The patient was followed up at the local hospital 1 month after surgery. ESWL was performed on the right upper ureter because the ultrasound indicated a few residual stones in the upper right ureter. Other laboratory tests: WBC 8.50 × 10^9^/L, N 0.75, HB 115 g/L, PLT 273 × 10^9^/L, Cre 86 μmol/L, urine WBC 4/μL, urine RBC 3/μL, PT 15.60 s, APTT 35.50 s, Fib 4.26 g/L. Three days after the operation, the urine and blood routine were not significantly abnormal, so it was decided to remove the right double J-tube, and the patient did not feel discomfort during and after the operation. The patient began to appear with these symptoms after the double J-tube removal. Therefore, the patient was immediately referred to our hospital. Physical examination showed pressure pain in the right upper abdomen, Murphy’s sign (+), no pressure pain, rebound pain, and muscle tension in the rest of the abdomen, no percussion pain in both kidney areas, and no significant abnormalities in other systemic examinations. Laboratory tests at admission showed: WBC 16.08 × 10^9^/L, N 0.82, HB 120 g/L, PLT 391 × 10^9^/L, Cre 131 μmol/L, Urine WBC 11/μL, Urine RBC 357/μL, ESR 120 mm/h, RF 143 IU/mL, ANA 1:40; Other laboratory results, including blood potassium, blood glucose, coagulation, liver function, and CRP levels were within normal limits. After transfer to our department, a computed tomography (CT) of the abdomen showed no significant abnormalities in both kidneys and bilateral ureters (Figure 1C). Other laboratory tests: WBC 8.83 × 10^9^/L, N 0.77, HB 97 g/L, PLT 373 × 10^9^/L, Cre 336 μmol/L, creatinine clearance 50.97 mL/min/1.73 m^2^, urine WBC 19/μL, urine RBC 14168/μL, PT 13.90 s, APTT 29.90 s, Fib 3.95 g/L. Four days later, the patient presented with significant gross hematuria with difficulty in urination, and a repeat abdominal CT showed multiple hemorrhages and hypodense lesions in the right kidney (Figure 2A), and the bladder was filled with a blood clot. The bladder clot removal and bilateral ureteral double-J tube placement were performed immediately. Intraoperatively, the right ureter was filled with a strip of the blood clot, and persistent hematuria emanated from the ureteral opening. With the patient’s and family’s consent, selective right renal artery embolization was performed immediately (Seldinger method, puncture of right femoral artery), and intraoperative angiography revealed multiple hemorrhages and pseudoaneurysm formation in the lower pole of the right kidney (Figure 2B), and the lower pole of the right renal artery was embolized using 2 platinum spring coils and 350 μm gelatin sponge particles. After embolization, the hematuria was relieved. Unfortunately, the patient reappeared with hematuria 3 days later. The relevant indexes were immediately reviewed: WBC 16.87 × 10^9^/L, N 0.86, HB 70 g/L, PLT 168 × 10^9^/L, urine WBC 0/μL, urine RBC 16,356/μL, PT 14.80 s, APTT 29.70 s, Fib 4.05 g/L. Abdominal CT showed hemorrhage and pneumatization in the right kidney and blood in the bladder, and it was considered that there was still active bleeding in the right kidney (Figure 2C). Since the patient and family strongly desired to preserve the kidney and refused to perform a right nephrectomy, selective right renal artery embolization was performed again (Seldinger method, puncture of right femoral artery). Intraoperative contrast reveals multiple hemorrhagic spots in the upper pole of the right kidney (Figure 2D), and the same was embolized using 2 platinum spring coils and 350 μm gelatin sponge particles in the upper pole artery of the right kidney. After two embolizations, the right kidney function was severely impaired, with less than 10% of normal kidney tissue preserved. Two hours after surgery, the patient developed sudden orthopnea, dyspnea, and coughing of pink foamy sputum, which was considered acute heart failure due to volume overload. No significant symptom improvement was seen after diuresis, volume vasodilation, and hemodialysis. At the same time, the patient presented with anuria and relevant laboratory tests: Cre 793 μmol/L, creatinine clearance 5.77 mL/min/1.73 m^2^, HB 82 g/L, NT-ProBNP 35,000 pg./mL. Therefore, he was transferred to the intensive care unit (ICU) during the night of the same day. After transfer, the patient was immediately treated with analgesia, sedation, absolute bed rest, and continuous hemodialysis for the ruptured right kidney bleeding. No recurrence of hematuria was seen during this period. Five days later, fungal spores and hyphae were found in the patient’s sputum, and fungal pneumonia was diagnosed. Related laboratory tests: WBC 13.23 × 10^9^/L, N 0.88, HB 59 g/L, PLT 203 × 10^9^/L, CRP 175.317 mg/L, PT 11.80 s, APTT 27.90 s, Fib 3.50 g/L, chest CT showed blurred lung texture in both lungs, and multiple patchy and striated high-density images were seen in both lung fields. On the sixth day in the ICU, the patient had a sudden onset of persistent lumbago on the left side. A repeat abdominal CT showed bleeding in both kidneys, with additional bleeding in the left kidney (Figure 3A). Other relevant laboratory tests: WBC 13.99 × 10^9^/L, N 0.90, HB 60 g/L, PLT 195 × 10^9^/L, urine WBC 3/μL, urine RBC 21,772/μL, Cre 373 μmol/L, creatinine clearance 14.36 mL/min/1.73 m^2^, Anti-neutrophil cytoplasmic antibody (ANCA) (−), PT 11.70 s, APTT 26.60 s, Fib 3.56 g/L; The patient’s bleeding in the left kidney was indicated for re-interventional embolization to stop the bleeding, but irreversible renal failure might occur after left kidney embolization, so the patient’s family refused interventional embolization. After up to 20 days of conservative treatment, the patient’s hemoglobin fluctuated at 52–72 g/L under constant red blood cell transfusion. Under continuous bedside ultrasound monitoring, the left perirenal hematoma gradually increased from 82 × 22 mm to 107 × 45 mm. The patient’s hemoglobin continued not to rise, and the left renal hematoma gradually increased. We considered that there was still active bleeding in the body. With the consent of the patient’s family, a left renal arteriogram was attempted on the patient (Seldinge method, puncture of the left femoral artery), a suspicious bleeding point in the left inferior pole artery was found intraoperatively (Figure 3B), therefore, 350 μm gelatin sponge particles were used to embolize this vessel. After up to 48 days of treatment, the patient’s condition gradually stabilized, with hemoglobin fluctuating at 60–76 g/L, creatinine fluctuating at 218–486 μmol/L, and the rest of the indicators in the normal range. Unfortunately, after three embolizations, the patient’s total renal function was severely impaired and irreversible, and he was dependent on hemodialysis to sustain his life. The patient was followed up 3 months after discharge and is currently on regular dialysis, able to take care of himself in daily life, with a daily urine output of about 600–1000 mL and creatinine fluctuating at 350–500 μmol/L. Ultrasound showed severe atrophy of the right kidney, mild atrophy of the left kidney, and no hydronephrosis in both kidneys. The diagnosis was chronic renal insufficiency. The patient is now undergoing regular monthly follow-ups and is considering a kidney transplant. The timeline from the first surgery to discharge is presented in Figure 4.

## Discussion

Spontaneous renal rupture is clinically rare and was first reported by Wunderlich in 1856 as idiopathic perirenal hematoma, also known as Wunderlich syndrome, characterized by spontaneous hemorrhage in the renal subcapsular and perinephric spaces in patients with no history of trauma (14, 15). Spontaneous rupture of the renal parenchyma or collecting system of the kidney is most often seen in pathologic kidneys, and associated pathologic manifestations include (1) Renal parenchymal lesions, such as tumors, inflammation, and tuberculosis; (2) Hydronephrosis or other lesions of the renal pelvis, such as renal pelvic calculi or narrowing of the ureteral junction of the renal pelvis; (3) Local necrosis due to compression of the renal pelvis wall by the calculi; (4) Secondary infection of the renal pelvis contributed to rupture of the renal pelvis (16–18). Some kidney-related operations may also contribute to the development of renal rupture. Renal rupture and bleeding after ureteroscopic holmium laser lithotripsy is a severe complication of ureteroscopic surgery and may be related to pathological changes in the patient’s kidney, intraoperative pelvic pressure elevation, and postoperative infection (9). Most of the complications of ESWL are rare, and ESWL is thought to induce renal capillary disruption, which causes ischemia and hypoxia. Increased formation of free radicals and subsequent damage to the kidney can occur in the reperfusion period (7, 19). The study by Chung et al. (7) reported that the kidneys of rats treated with ESWL all had more severe wear and inflammation, and their damage increased with the number of treatments. Clinical studies have also consistently concluded that the severity of kidney damage is closely related to shock-wave energy, shock frequency, etc. (3, 20). In addition, hypertension, diabetes, obesity, autoimmune diseases, and coagulation disorders are all risk factors for forming perirenal hematoma (21). Therefore, ESWL should be performed cautiously in patients with combined underlying disease and when the underlying disease is effectively controlled and the patient is fully informed of the associated complications and risks.

Spontaneous renal rupture lacks specific clinical signs and symptoms, and the characteristic Lenk triad of flank pain, a flank mass, and hypovolemic shock can be observed in only 20% of patients (22). The definitive diagnosis relies on CT, magnetic resonance imaging, and ultrasound (23). Among other things, CT can quickly help identify acute abdominal diseases such as cholecystitis, pancreatitis, appendicitis, urinary stones, renal rupture and splenic rupture. In addition, in the case of renal rupture bleeding, CT not only shows the extent of renal bleeding quickly and accurately, but also elucidates the underlying etiology of the bleeding and excludes secondary causes (24, 25).

Treatment of spontaneous renal rupture should be based on the nature of the primary disease, the anatomical location of the rupture, the severity of the rupture, and the hemodynamic stability of the patient, including conservative and surgical treatment (24). The treatment is mainly conservative for those with stable vital signs and a strong desire for renal preservation, and SAE is feasible if necessary. SAE has been demonstrated to be effective and safe in treating renal hemorrhage from all causes, and its value in controlling spontaneous ruptured renal hemorrhage and avoiding nephrectomy is significant (26, 27). However, SAE has a high re-embolization rate for some renal secondary ruptured bleeding lesions, and patients will face partial renal parenchymal ischemia and subsequent loss of its function (28, 29). However, the study by Huber et al. (30) showed that since there were equal success rates for the initial and the repeated intervention, re-intervention was justified when the clinical course allowed. Therefore, selective renal artery embolization should be treated with caution. It has also been suggested that angiography should be performed in cases of idiopathic renal hemorrhage to clarify the diagnosis and embolize the treatment and avoid unnecessary nephrectomy (23, 24). In patients with failed conservative treatment and recurrent bleeding, anti-shock therapy should be actively administered along with surgery, and exploratory surgery should be used as a life-saving measure. However, radical nephrectomy is unavoidable in such conditions.

In this case, the patient had no previous history of underlying disease or trauma, and no significant abnormalities of both kidneys were seen on abdominal CT after transfer to our department. The patient underwent ureteroscopic lithotripsy at an outside hospital, and his procedure was successful with no postoperative complications. Holmium laser does not cause scattered or direct damage to the renal parenchyma during ureteroscopic lithotripsy. The patient’s clinical symptoms started after removing the double J-tube shortly after extracorporeal shock wave lithotripsy. It was considered that the double J tube could play a triple role of prop up, drainage, and dilation in the ureter, which facilitated calculi expulsion and effectively reduced the incidence of related complications (31, 32). This patient had a double J-tube removed 3 days after ESWL and had a renal rupture at 3 weeks postoperatively. The patient had no underlying diseases such as hypertension, diabetes, hematologic diseases, etc. And we did relevant immunological tests to exclude spontaneous renal rupture due to autoimmune pathology. Therefore, we considered that the patient developed a delayed right kidney rupture after ESWL. Because the patient’s ureteroscopic lithotripsy and ESWL were not performed at our institution and the initial consultation was in the Department of Hepatobiliary Surgery, there may have been a lack of standardization of specialty care and management, which may have contributed to the patient’s delayed renal rupture. Thus, for patients with a double J-tube placed in the body, the removal time can be extended appropriately after ESWL. In the event of rebleeding from the patient’s right kidney, we recommend radical nephrectomy for such uncontrollable bleeding to achieve hemostasis (33). However, the patient strongly desired to preserve his kidney and refused surgical intervention. The result of multiple embolisms is ischemic kidney necrosis leading to acute renal failure. After being transferred to the intensive care unit, the patient developed an uncontrollable systemic infection and acute renal failure. Although we had started relevant antibiotics and continuous hemodialysis in the early stage, the left kidney developed a spontaneous ruptured hemorrhage. During this period, continuous monitoring of patients’ coagulation function showed no obvious abnormalities. It is considered that spontaneous left kidney rupture may be associated with a long-term systemic inflammatory response and edema of the renal tissue due to renal failure. According to the literature, patients with renal failure have a higher risk of spontaneous renal rupture, especially in patients receiving hemodialysis, because using anticoagulants may increase the risk of renal rupture (10, 13). Although infection is not a common risk factor for spontaneous renal rupture, we should consider this risk factor comprehensively in appropriate conditions (22). Given the patient’s complicated condition, we did not consider surgical intervention but mainly conservative treatment. We continuously monitored the changes in hemoglobin and left renal hematoma size and found that the left renal hematoma was slowly increasing. Therefore, we attempted to perform left renal arteriography to clarify the presence of active bleeding in the left kidney, and embolization of the lower pole vessels of the left kidney was performed to stop the bleeding according to the intraoperative situation.

## Conclusion

Through this case report, we have the following clinical experience for reference: (1) When performing ESWL, special attention should be paid to the shock wave energy and the number of shocks, and the degree of postoperative renal function damage is directly proportional to it. (2) For patients with a double J tube placed in their bodies, the extubation time after ESWL can be extended appropriately according to the presence or absence of fever and lumbar signs. (3) Based on heavy systemic infection and acute renal failure, the likelihood of spontaneous renal rupture increases significantly, so the reasonable use of anticoagulants and early infection control are vital for the overall regression of the patient’s condition. (4) In patients with renal failure and bleeding from ruptured kidneys, early hemostasis is more critical than salvage of renal function; therefore, early intervention is crucial for patient prognosis.

## Ethics statement

The studies involving humans were approved by the Affiliated Hospital of Zunyi Medical University. The studies were conducted in accordance with the local legislation and institutional requirements. The participants provided their written informed consent to participate in this study. Written informed consent was obtained from the individual(s) for the publication of any potentially identifiable images or data included in this article.

## Author contributions

WT and DY were the main contributors to the writing and revision of this manuscript. TW provided the initial idea, financial aid, and the study design and performed some crucial recommendations. GL provided essential suggestions for the discussion section. All authors read and approved the final version of the manuscript.
